# Bone Proteomics Method Optimization for Forensic Investigations

**DOI:** 10.1021/acs.jproteome.4c00151

**Published:** 2024-04-15

**Authors:** Luke Gent, Maria Elena Chiappetta, Stuart Hesketh, Pawel Palmowski, Andrew Porter, Andrea Bonicelli, Edward C. Schwalbe, Noemi Procopio

**Affiliations:** † School of Law and Policing, Research Centre for Field Archaeology and Forensic Taphonomy, University of Central Lancashire, Preston PR1 2HE, United Kingdom; ‡ Department of Biology, Ecology and Earth Sciences (DiBEST), University of Calabria, Arcavacata di Rende 87036, Italy; § School of Medicine, University of Central Lancashire, Preston PR1 2HE, United Kingdom; ∥ NUPPA Facility, Medical School, Newcastle University, Newcastle Upon Tyne NE1 7RU, United Kingdom; ⊥ Department of Applied Sciences, Northumbria University, Newcastle Upon Tyne NE1 8ST, United Kingdom

**Keywords:** bone proteomics, protein extraction, mass spectrometry, forensic science, acquisition modes

## Abstract

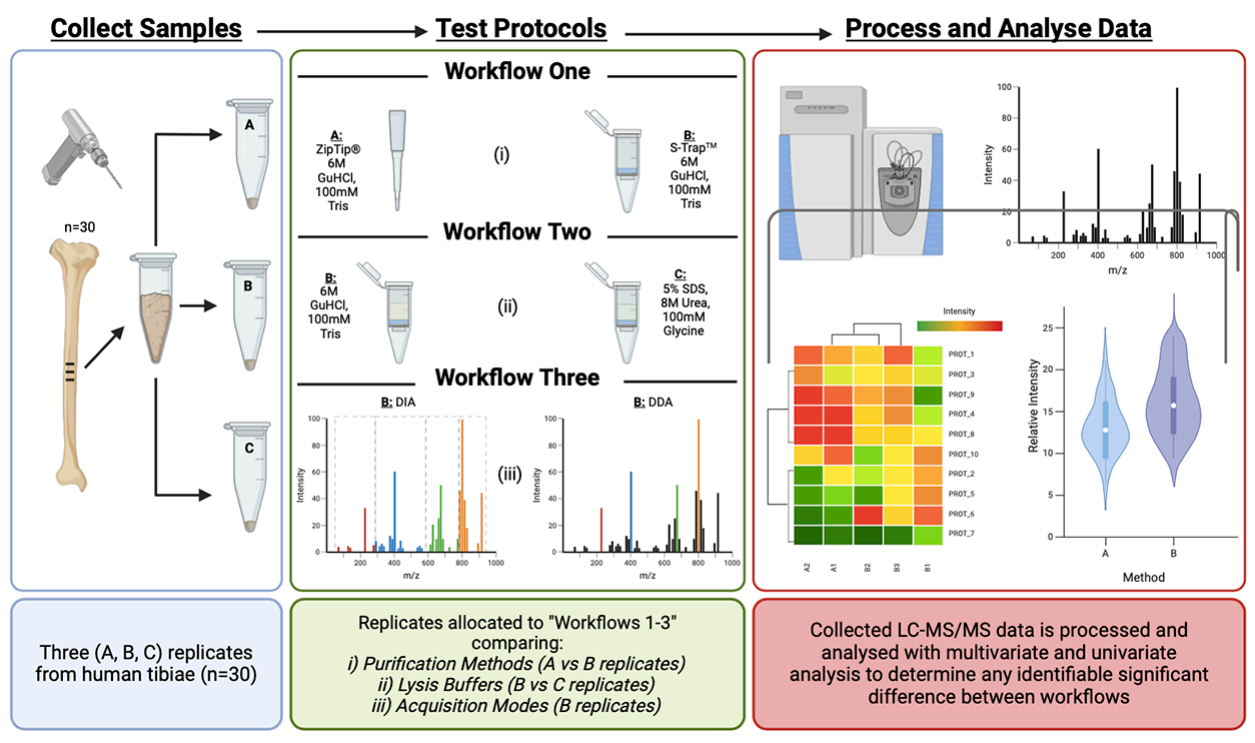

The application of proteomic analysis to forensic skeletal remains has gained significant interest in improving biological and chronological estimations in medico-legal investigations. To enhance the applicability of these analyses to forensic casework, it is crucial to maximize throughput and proteome recovery while minimizing interoperator variability and laboratory-induced post-translational protein modifications (PTMs). This work compared different workflows for extracting, purifying, and analyzing bone proteins using liquid chromatography with tandem mass spectrometry (LC–MS)/MS including an in-StageTip protocol previously optimized for forensic applications and two protocols using novel suspension-trap technology (S-Trap) and different lysis solutions. This study also compared data-dependent acquisition (DDA) with data-independent acquisition (DIA). By testing all of the workflows on 30 human cortical tibiae samples, S-Trap workflows resulted in increased proteome recovery with both lysis solutions tested and in decreased levels of induced deamidations, and the DIA mode resulted in greater sensitivity and window of identification for the identification of lower-abundance proteins, especially when open-source software was utilized for data processing in both modes. The newly developed S-Trap protocol is, therefore, suitable for forensic bone proteomic workflows and, particularly when paired with DIA mode, can offer improved proteomic outcomes and increased reproducibility, showcasing its potential in forensic proteomics and contributing to achieving standardization in bone proteomic analyses for forensic applications.

## Introduction and Background

Since their inception, untargeted proteomics methodologies have been widely applied across diverse scientific domains, prominently in forensic science. The integration of bottom-up proteomic approaches in forensic investigations has proven instrumental in uncovering novel biomarkers with significant implications for the precise determination of post-mortem interval (PMI),^1−5^ assessment of age-at-death,^6,7^ identification of body fluids,^8,9^ and establishment of identity.^10^

Forensic specimens encompass a variety of distinct tissues and fluids including bones. Proteomic analyses in forensics have enabled a first understanding of the molecular changes associated with cadaveric decomposition and how intrinsic and extrinsic factors can influence this process,^5,7,11^ especially on long-term PMIs (i.e., > 2 years) with partially or fully skeletonized remains.^6^ For these situations, teeth or bones are commonly used as the starting material for analysis, necessitating specific adjustments to conventional proteomic protocols, such as the addition of demineralization steps to extract the proteome from the mineral hydroxyapatite matrix inherent to skeletal tissue.^12^

To apply this methodology to forensic casework, first, it is crucial to optimize and standardize protocols to allow ideally for high-throughput analyses, decreased operator bias and batch effects, increased protein recovery rate, and reduced laboratory-induced post-translational modification (PTMs) rates and overall protocol length and complexity. Sample preparation lays, in fact, the foundation for accurate and reproducible results in downstream analyses of bone proteomic data. Standard steps in bone extraction protocols typically include bone demineralization, protein denaturation, reduction, alkylation, digestion, purification, concentration, desalting, and peptide reconstitution prior to liquid chromatography with tandem mass spectrometry (LC–MS)/MS runs.^13,14^ The most commonly used procedures for bottom-up proteomics of bone samples following demineralization include Solid-Phase-enhanced sample preparations (SP3), in-StageTip (iST) commercial products (such as ZipTip and StageTip), and filter-aided sample preparation (FASP) protocols.^15−17^

Protocols for the applications of proteomics to bone in archeological and paleontological contexts have been deeply studied and adapted to maximize protein and peptide identification and coverage from samples that typically have low and degraded protein content.^13^ However, less investigation has been done with specific reference to forensically relevant bone material. Forensic specimens (i.e., <100 years since deposition) may have better protein preservation compared to historical specimens (defined as ≥100 years), due to the shorter chronological age of such specimens.^18^ However, exposure to environmental factors such as temperature fluctuations, wet and dry cycles, humidity, and sunlight can significantly affect the biomolecular preservation of forensic specimens, particularly when they are exposed on the surface for relatively long periods of time or buried in various types of coffins.^19^ There is a currently unmet need to optimize protocols for forensic specimens by testing existing workflows developed for forensic and/or archeological samples to ultimately propose novel and improved protocols specifically tailored to forensics.

The first and only method developed so far specifically for PMI estimation from bone protein extracts of forensic interest was reported by Procopio and Buckley.^14^ This protocol maximizes the diversity of the extracted proteome while reducing artificially induced PTMs (such as deamidations), which result from harsh chemical or physical processing of the samples during their extraction. After bone demineralization and protein denaturation, this protocol uses ZipTip, a type of iST that offers single-step desalting, concentration, and purification.^14^

Since its publication, this extraction method has been routinely applied in forensic bone proteomics.^4−6,20^ However, this approach is technically demanding, difficult to adapt to large sample cohorts (i.e., >100), and sample loss can occur due to sample transfer between vessels.^21−23^

We tested a novel sample preparation for forensic bone proteomics using the S-Trap technology. This approach aims to increase reproducibility between extractions by creating a fine particulate suspension that is more accessible for rapid enzymatic action.^24−26^ Importantly, a single tube is used for sample cleaning, incubation, and digestion, minimizing sample loss and reducing the time taken for the sample preparation tube.^24^ A previous study using HeLa cells reported that an optimal lysis buffer was either 5% sodium dodecyl sulfate (SDS) or 4% SDS with 0.1 M dithiothreitol,^25^ which is consistent with manufacturer guidelines.^24^ However, S-Trap, to the authors’ knowledge, has not previously been used in bone proteomics protocols for forensic applications, although it has been already applied in paleontological and archeological investigations.^27,28^

There is increasing interest in the use of the data-independent acquisition (DIA) mode compared to the more commonly used data-dependent acquisition (DDA) mode for forensic bone proteomics.^29^ DDA analyses digest samples into peptides, which are then visualized by tandem MS/MS spectra; these are matched to a spectral database for fragment identification. This technique favors the identification of high-abundance peptides,^30^ and it is challenging to reproducibly quantify low-abundance peptides using this approach.^31^ In contrast, DIA analysis identifies all peptides within a sliding mass-to-charge (*m*/*z*) window.^29,32^ This results in accurate peptide quantification without being limited to profiling predefined peptides of interest, which increases data reproducibility,^33,34^ also between different laboratories. A further challenge with DIA analyses is data processing. The complex MS2 spectra require specific analytical tools. The introduction of software such as Spectronaught, DIA-NN, OpenSWATH, FragPipe, and Skyline has enabled the deconvolution of complex MS/MS spectra, allowing for more accurate peptide quantitation.^35^

Herein, this study aimed to optimize the use of S-Trap for forensic bones to increase extraction reproducibility and throughput, while comparing DDA and DIA acquisition modes for optimal forensic bone proteomics methods.

## Methods and Materials

### Sample Collection and Subsample Preparation

**Figure 1:**
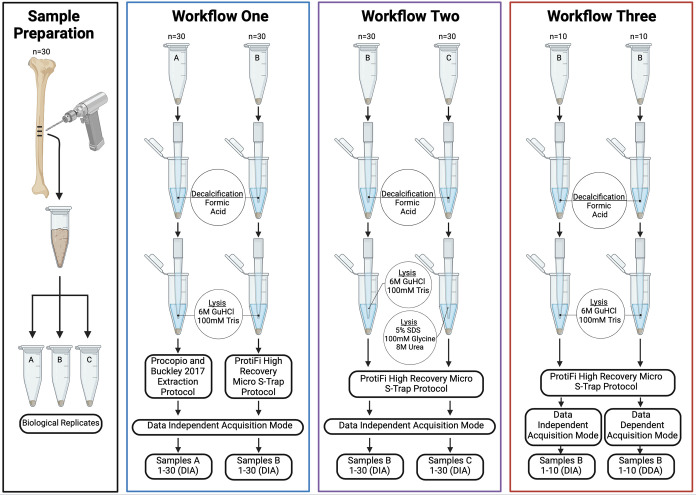
Outline of the three study workflows investigated including the number of samples (1–30), in which biological replicates are compared, extraction protocol, and mass spectrometric acquisition mode. Further details of the extraction protocols are outlined in Table S1. “Workflow One” is a comparison between protein extraction techniques. “Workflow Two” is a lysis buffer comparison using the novel S-Trap Protocol. “Workflow Three” is an investigation between DDA and DIA acquisition modes.

This study was approved by the Research Ethics Committee IRAS (ref 22/NI/0118) and the University of Central Lancashire Ethics Committee (ref SCIENCE 0223). The midshaft of the tibia of 30 human donors of known age (33–93 years) was sampled by the University of Sam Houston—Southeast Texas Applied Forensic Science Facility (STAFS) at various post-mortem intervals (189–2237 days) using bleached diamond cutting blades and a Dremel. The midshaft tibia was chosen for subsampling based on previous investigations into the biomolecular changes of human skeletal remains.^4,6,7^ Specifically, window cuts of approximately 1 cm^3^ were taken and shipped to the University of Central Lancashire for further processing. Bone powder (approximately 25 mg) was taken from each subsample using bleached dental drill bits and a Dremel, by creating transverse parallel lines across each fragment in triplicate biological replicate (“A,” “B,” and “C”; justified as biological replicates as established in previous research such as in ref (7) samples; Figure 1 details the use of replicate samples for the three investigated workflows.

### Protein Extraction Experimental Workflows

The three main experimental workflows investigated are detailed in Figure 1 and Table S1. Workflow One compared the optimized protocol proposed by Procopio and Buckley^14^ and an adapted version of the S-Trap micro-spin column digestion protocol that includes the same lysis buffer adopted by Procopio and Buckley (6 M guanidine hydrochloride (GuHCl) and 100 mM Tris) and analyses conducted in the DIA mode. This was done on two sets of biological replicates, sets A and B for 30 human samples. Workflow Two was a comparison of lysis buffer using the adapted S-Trap micro-spin column digestion protocol, where 6 M GuHCl/100 mM Tris buffer is compared to the lysis buffer recommended by the manufacturers (5% SDS, 100 mM glycine, and 8 M urea). Analyses were conducted in the DIA mode. This was done on B and C sets of biological replicates for 30 human samples. Workflow Three is a comparison between the DIA and DDA modes only, using 10 samples from Workflow Two-set B (samples extracted using the S-Trap protocol and GuHCl/Tris as the lysis buffer), both run in the DIA and DDA modes.

Abbreviations:AMAC: ammonium acetateFA: formic acidGuHCl: guanidine hydrochlorideTris: tris bufferSDS: sodium
dodecyl sulfateDTT: dithiothreitolIAM: iodoacetamideTFA: trifluoroacetic acidTEAB: tetraethylammonium bromideACN: acetonitrileMaterial sources are in Supporting Information

### LC/MS-MS Analysis

Samples were resuspended in 3% ACN/0.5% FA and analyzed by LC–MS/MS using an Ultimate 3000 Rapid Separation LC (RSLC) nano-LC system (Thermo Corporation, Sunnyvale, CA) coupled with an Exploris 480 Quadrupole-Orbitrap Mass Spectrometer (Thermo Fisher Scientific, Waltham, MA). A volume equivalent to 1 ng of peptides (per injection) was first loaded onto an Acclaim PepMap 100 C18 LC Column (5 mm Å ∼ 0.3 mm i.d., 5 μm, 100 Å, Thermo Fisher Scientific) at a flow rate of 10 μL/min maintained at 45 °C and separated on an EASY-Spray reverse phase LC Column (250 mm Å ∼ 75 μm diameter (i.d.), 2 μm, Thermo Fisher Scientific, Waltham, MA) using a 60 min gradient from 97% A (0.1% FA in 3% DMSO) and 3% B (0.1% FA in 80% ACN 3% DMSO) to 35% B at a flow rate of 250 nL/min. The separated peptides were then analyzed with either data-dependent (DDA) or data-independent (DIA) acquisition according to the specified workflows (Figure 1).

For DDA acquisition in the full scan mode, the MS resolution was set to 60,000 with a normalized automatic gain control (AGC) of 300%, a maximum injection time of 50 ms, and a scan range of 400–1600 *m*/*z*. The top 20 most abundant ions were selected for MS/MS, with a normalized collision energy level of 30% performed at 15,000 MS resolution with an AGC of 100% and maximum injection time set to “Auto.” The isolation window was set to 1.4 *m*/*z*. Dynamic exclusion was employed after one repeat scan (i.e., two MS/MS scans in total) was acquired, with the precursor being excluded for the subsequent 35s.

For DIA acquisition in the full scan mode, the MS resolution was set to 60,000 with a normalized automatic gain control (AGC) of 100%, dynamic maximum injection time, and a scan range of 390–1010 *m*/*z*. %. DIA MS/MS were acquired with 45 variable width windows covering 410–1183 *m*/*z*, at 15,000 resolution, dynamic maximum injection time with an ACG target of 1000%, and a normalized collision energy level of 30%.

### Data Analysis

The acquired data from the workflows that utilized DDA mode were analyzed with MaxQuant version 2.0.3.0 [default settings, variable modifications: deamidation (NQ) and oxidation (M); fixed modification: carbamidomethyl (C)] and for workflows that employed DIA mode, DIA-NN (version 1.8) was used (Library free, default settings, with deep learning-based spectra RTs and IMs prediction; variable modifications: deamidation (NQ), acetyl (Protein N-term), and oxidation (M); fixed modification: carbamidomethyl (C), N-term M excision). All analyses were searched against the human proteome database (Uniprot: UP000005640, version 10/28/2021), and subsequent data processing was carried out in R v4.3.0.

### Bioinformatic Preprocessing

**Figure 2:**
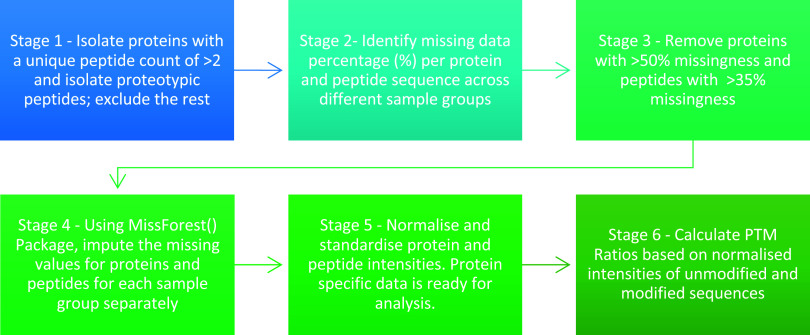
Summary outline flowchart of DDA and DIA proteomics data analysis following MaxQuant and DIA-NN workflows. Standardization of data occurred only for Workflow 3.

Data aggregation of DIA-NN and MaxQuant outputs was performed by using R and R Studio (Figure 2). Post-translational modifications (PTMs) and protein abundance were assessed. For “Workflow 3” (Figure 1), PTM ratios were unavailable due to the lack of “matching” comparable modified sequences between both the DDA and DIA mode proteome data sets.

Missing data is common in proteomics and is dependent on sample quality, source, and preprocessing analytical choices. The MissForest R package was chosen to impute missing data (further information on choosing an imputation algorithm is found in the Supporting Information and illustrated in Figure S1) based on performance metrics (root mean squared error and Pearson correlation coefficients between real and imputed values) when compared to three other imputation methods (further information on this comparison and performance metrics are in the Supporting Information, Figures S2–S4 and Tables S2–S4). Rather than focusing on proteins and peptides with complete data, the inclusion of imputation allowed for a full investigation of identified species (both proteins and peptides). Acceptable missingness cut-offs for determining whether to attempt imputation for missing proteins (e.g., those proteins for which the data for specific samples is empty) and peptides were empirically determined by artificially removing data (from 30 to 50% at 5% intervals) and assessing concordance between true and imputed data values (further information on choosing a cutoff threshold can be found in the Supporting Information).

Protein and peptide data were normalized using log2 transformation, and standardization of the data in Workflow 3, which compared data from different acquisition modes and different analytical runs, was performed by z-score scaling per individual sample. Z-score scaling for the comparison between “Workflow 1” and “Workflow 2” was not necessary since the workflows were part of the same analytical run and were measured with the same acquisition mode.

PTM ratios were calculated from the total relative abundance of the modified state of a sequence and the total relative abundance of the corresponding peptide sequence (example shown in Mizukami et al.^11^).
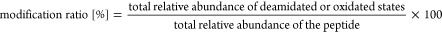


Thresholds for applying imputation differed between proteins and peptides; for proteins, those beyond a certain level of missingness were removed; for peptides, if any of the identified sequences for a peptide, whether modified or not, exceeded the missingness cutoff, then that peptide was not analyzed. The modification ratio would be inaccurate if not all available sequences for a peptide were used; if all sequences did not pass the cutoff, then it was excluded entirely.

PTMs of interest and set as variable modifications were deamidated asparagine (N), deamidated glutamine (Q), and oxidated methionine (M) due to their association with *in vivo* and post-mortem aging in bones and their consequent importance in the analysis of forensic samples. Acetylation (N-term) is added by default in the DIA-NN and MaxQuant software and is not observed here as a forensically relevant PTM.

### Statistical Analysis

Protein and peptide data were analyzed using principal component analysis (PCA) and intensity heatmaps with Euclidean hierarchical clustering, followed by univariate statistical analyses. All statistical analysis was conducted in R v4.3.0.

## Results

### Analysis for Workflow One: Procopio and Buckley vs S-Trap Protocol

The initial number of proteins identified using the Procopio and Buckley protocol was 109, whereas 138 were identified with the S-Trap GuHCl/Tris protocol. Following data preprocessing and cleanup (removal of specific proteins that exhibited too much absent data across the sample set), 76 proteins were retained in the Procopio and Buckley group and 86 were retained in the S-Trap GuHCl/Tris group. Among those, 66 proteins and 135 peptides were identified and retained in both protocols after data missingness removal and imputation. Fourteen peptides with defined PTMs were identified in both groups.

Results showed a higher percentage of missing data for the Procopio and Buckley protocol (27.3%) as opposed to the S-Trap group (18.2%) (Figure S2), although the difference was not significant (*p* > 0.05, Wilcoxon-signed rank test); there were slightly lower levels of deamidations in the S-Trap group (47.5%) in comparison with the Procopio and Buckley group (48.8%) and similar levels of oxidations (50% in the S-Trap group and 49.7% in the Procopio and Buckley group).

Proteome coverage for Workflow One revealed a significant difference (*p* < 0.001) between the groups of interest at a group comparison level (proteome coverage is defined in the Supporting Information). Further details on which proteins were classified as significantly different between groups at an individual protein level are outlined in Tables S5.1 and S5.2.

**Figure 3:**
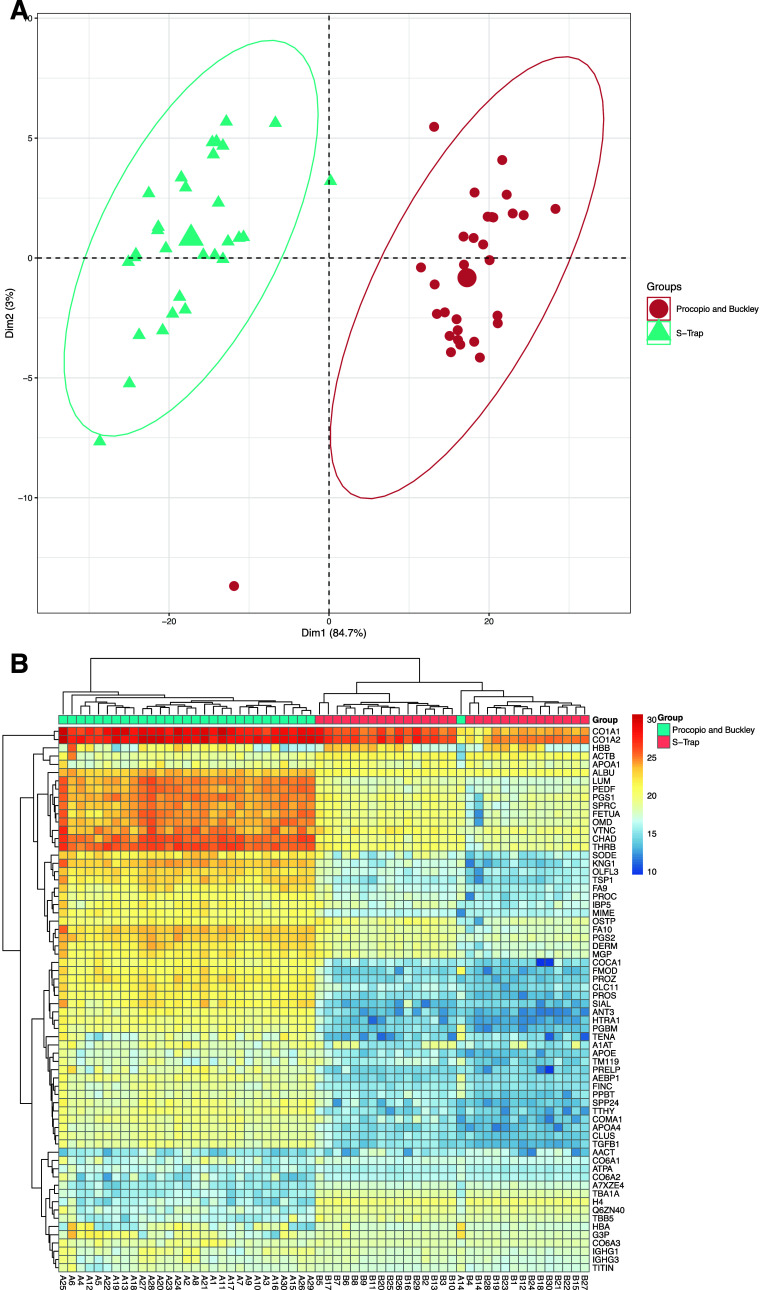
(A) Principal component analysis (PCA) of 66 proteins found within the Procopio and Buckley versus S-Trap experiment subgroups for each of the human skeletal tibiae specimens. Axes 1 and 2 explain 87.7% of the variance. (B) Heatmap between Workflow One subgroups “Procopio and Buckley” and “S-Trap” for proteins. Scale is in normalized abundance.

The PCA and heatmap for the multivariate analysis showed the presence of workflow-specific groups with a high dissimilarity compared to each other based on relative abundances (Figure 3A,B). When comparing the groups, overall, the Procopio and Buckley method demonstrated higher protein relative abundances (Figure 3B), with 58 out of 66 proteins being significantly different between the workflows (Wilcoxon-signed rank test, *p* < 0.05; Table S6.1). Of these 58 proteins, 52 were higher in abundance for the Procopio and Buckley group, with 4 higher in abundance in the S-Trap group.

**Figure 4:**
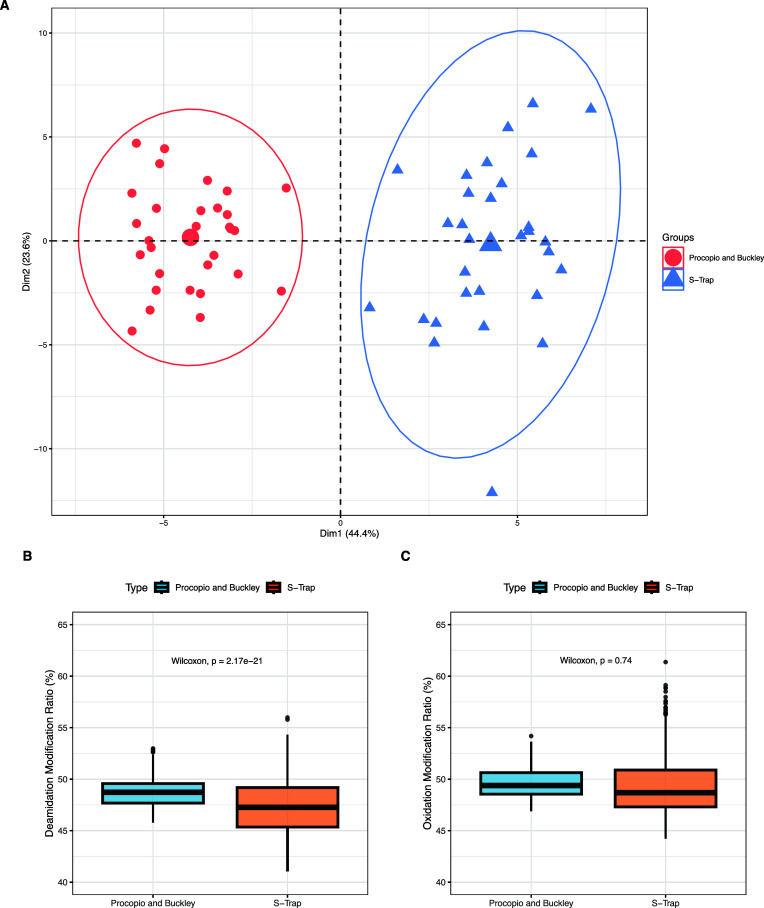
(A) PCA of 14 peptides found within the ZipTip versus S-Trap experiment subgroups for each of the human skeletal tibia specimens that have had PTM to specific amino acid residues. Axes 1 and 2 explain 68% of the variance. (B) Boxplot of deamidation ratios (%) in the sample groups of Procopio and Buckley vs S-Trap. (C) Boxplot of oxidation ratios (%) in the sample groups of Procopio and Buckley vs S-Trap.

At a peptide level when investigating differences in PTMs, the PCA of the matched modified (specific to deamidation and oxidation) peptides shows a clear protocol-dependent separation (Figure 4A) with 11 out of 14 modified peptides having significant differences in their relative abundances (Wilcoxon-signed rank test, *p* < 0.05; S5.2). Of those 11 peptides, 9 had a higher modification ratio in the Procopio and Buckley group, and only 2 had a higher ratio in the S-Trap group. By looking at specific modifications, only deamidated peptides were significantly different (*p* < 0.001) (Figure 4B), whereas the oxidated ones were not (*p* > 0.05, Figures 4C and S5). The differences in mean and range of modification ratios for the S-Trap group compared to the Procopio and Buckley group were greater for the deamidated peptides than for the oxidated peptides (Figure 4B). However, statistical inference here was limited by the small number of peptides being compared in terms of distribution (nine deamidated and five oxidated peptides specifically).

### Analysis for Workflow Two: S-Trap Solubilization Detergents

The two lysis solutions used with the S-Trap protocol resulted in a similar initial number of proteins identified (GuHCl/Tris solution = 138 proteins; SDS solution = 141 proteins). Following data preprocessing and cleanup (removal of specific proteins that exhibited too much absent data across the sample set), 113 proteins were found in the GuHCl/Tris group and 119 were found in the SDS group. Among those, 112 proteins and 385 peptides were identified in both protocols and retained after data missingness removal and imputation. Twenty-one peptides exhibited the PTMs of interest in both groups.

The percentage of missing data for the two protocols was similar (GuHCL/Tris—18%; SDS—16.1%; *p* > 0.05, Wilcoxon-signed rank test; Figure S3), as well as levels of deamidations and oxidations in the GuHCl/Tris group (48.6 and 51.1% respectively) and in the SDS group (48.7 and 51.3%, respectively).

Proteome coverage for Workflow Two revealed a significant difference between the groups of interest (*p* < 0.001) at a group comparison level; further details of which proteins were classified as significantly different between the compared groups at an individual protein level are outlined in Tables S5.3 and S5.4.

**Figure 5:**
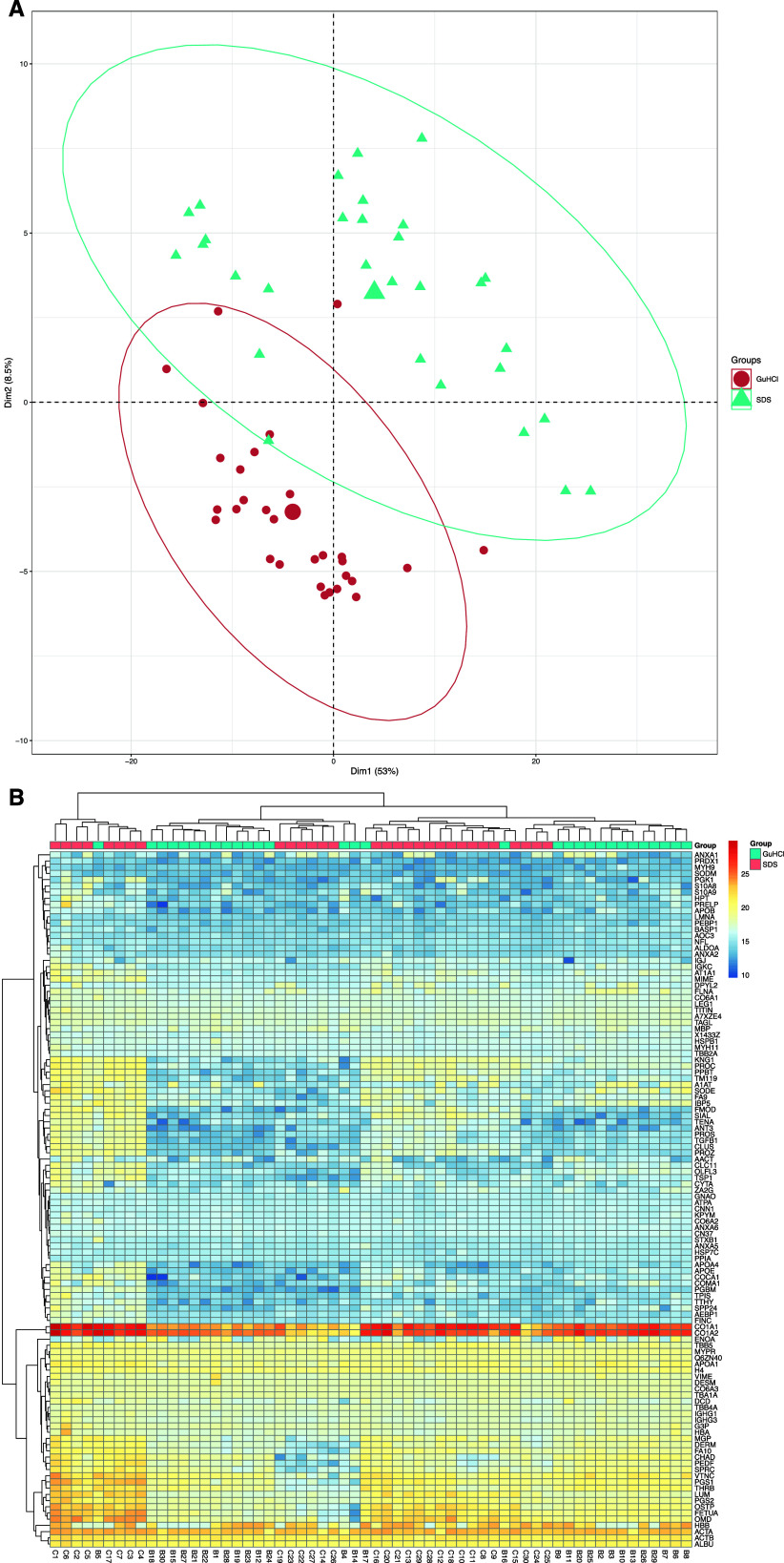
(A) PCA of 112 proteins found within the GuHCl/Tris versus SDS experiment subgroups for each of the human skeletal tibiae specimens. Axes 1 and 2 explain 61.5% of the variance. (B) Heatmap between Workflow Two subgroups “GuHCl/Tris” and “SDS” for proteins. Scale is in normalized abundance (i.e., unitless).

The PCA showed an overall difference between buffers at the protein level (*p* < 0.001) (Figure 5A). However, the heatmap showed that the majority of identified proteins across buffers were similar (Figure 5B). Indeed, the relative abundance of 78 of the 112 proteins was not significantly different between the groups (Table S6.3). Of those 34 proteins that were significantly different, 30 were higher in abundance in the SDS group compared to the 4 higher in abundance in the GuHCl/Tris group.

**Figure 6:**
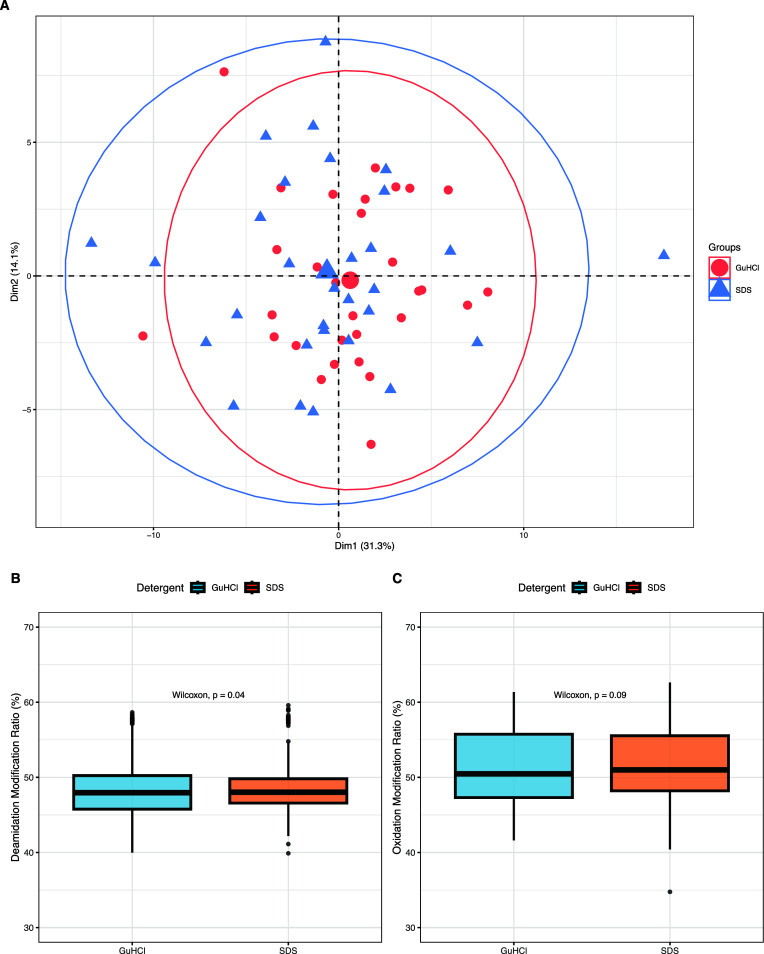
(A) PCA of 21 peptides found within the GuHCl/Tris versus SDS experiment subgroups for each of the human skeletal tibiae specimens that have had PTM to specific amino acid residues. Axes 1 and 2 explain 45.4% of the variance. (B) Boxplot of deamidation ratios (%) in the sample groups of GuHCl/Tris vs SDS. (C) Boxplot of oxidation ratios (%) in the sample groups of GuHCl/Tris vs SDS.

At a peptide level when investigating differences in PTMs, the PCA of the deamidated and oxidated peptides shows that the SDS cohort is comparable to the GuHCl/Tris cohort (Figure 6A) with 11 out of the 21 modified peptides having no significant difference in their relative abundances. At a group level, there is a significant difference for deamidation modification ratios (*p* < 0.05 Figures 6B and S6) but not for oxidation modification ratios (*p* > 0.05 and Figures 6C and S6).

### Analysis for Workflow Three: DDA vs DIA Acquisition Modes

The two acquisition modes resulted in a large difference in the initial number of unique proteins identified (DDA = 49 proteins; DIA = 143 proteins). Following data preprocessing and cleanup (removal of specific proteins that exhibited too much absent data across the sample set), 119 proteins were found in the DIA group and 28 were found in the DDA group. Among those, only a limited number of proteins (*n* = 22) were identified in both acquisition modes and retained after data missingness removal and imputation. Peptide PTM ratios were not investigated in this workflow due to the lack of identified communal modified peptides. DIA identified a higher number of proteins as well as detecting different sets of identifiable peptides; in contrast, the majority of peptides found using DDA were either unmodified or contained high amounts of missing data. It should be noted that unlike workflows one and two, which were acquired solely in the DIA mode and analyzed with the same MS software DIA-NN, the raw data for the DDA mode was analyzed using MaxQuant.

The percentage of missing data for the two acquisition modes was largely different (DDA mean missingness DDA 42.2 vs 6.5% for DIA; *p* < 0.001; Figure S4).

Proteome coverage for Workflow Three revealed no statistically significant differences between the groups of interest; further details of which proteins were classified as significantly different between the compared groups at an individual protein level are outlined in Tables S5.5 and S5.6.

**Figure 7:**
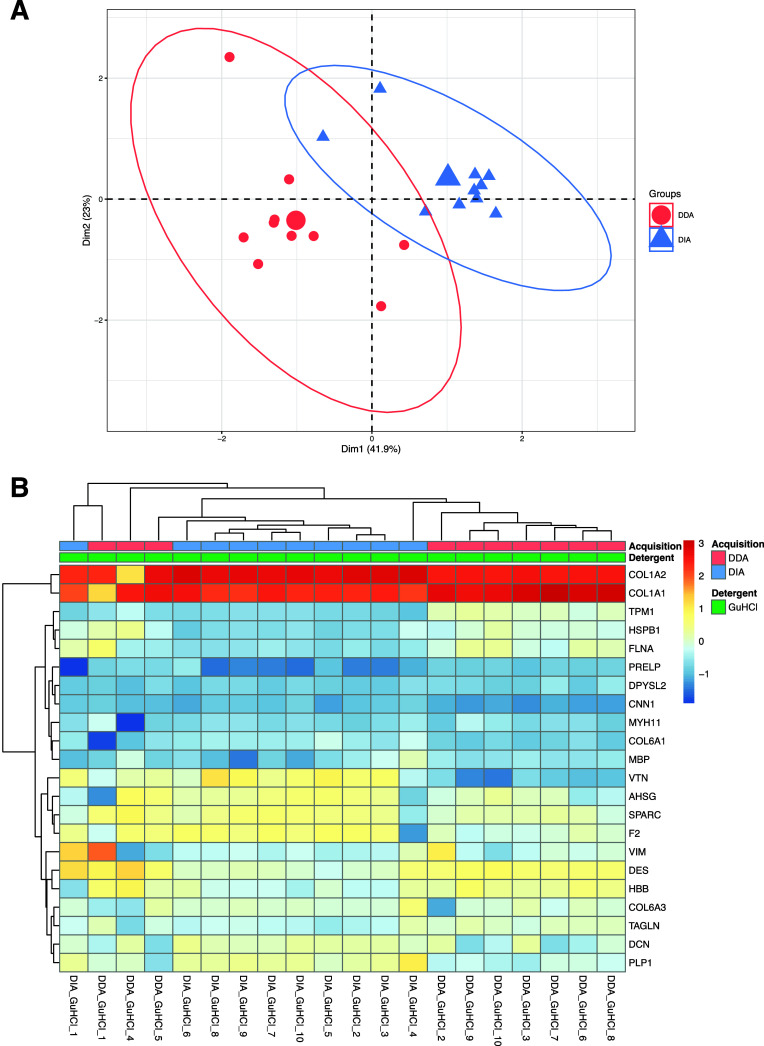
(A) PCA of 22 proteins found within the DDA versus DIA experiment subgroups for each of the specimens. Axes 1 and 2 explain 64.9% of the variance. The centroids are represented by the larger circle and triangle icons. (B) Heatmap with Euclidean hierarchical clustering between Workflow Three subgroups “DDA” and “DIA” for proteins.

The PCA revealed significant differences between the groups, particularly at the protein level (Figure 7A). Notably, both modes demonstrate consistent identification of the same highly abundant proteins, with differences emerging when observing the lower-abundance proteins, as further confirmed by the heatmap (Figure 7B). Twelve out of 22 proteins identified in both modes showed a significantly different relative abundance between the two groups (*p* < 0.05, Table S6.5). Of these 12 proteins that were significantly different, 6 were higher in abundance in the DDA group and the other 6 were higher in abundance in the DIA group. These findings hold practical implications for choosing the acquisition mode in investigations, particularly in obtaining a larger cohort with a larger dynamic range.

## Discussion

The aim of this multilevel study was to investigate different sample preparation protocols of forensic bone material coupled with two data acquisition modes to assess their ultimate suitability for applications in forensics.

This direct comparison between methods has led to the finding that novel sample preparation techniques and improved analytical strategies can ultimately provide optimized bone proteomics methods that are able to offer a few advantages in comparison with the previously optimized method by Procopio and Buckley. From the results obtained, it appears that coupling the S-Trap technology with the DIA mode can offer improved protein identification and reduced levels of PTMs in comparison to the Procopio and Buckley protocol. Additionally, using the S-Trap workflow, it is possible to conduct high-throughput studies and minimize extraction errors caused by the operator by increasing robustness. The following discussion goes into detail and suggests how this comparison has offered a platform for the future development of a high-throughput bone proteomic workflow with available technologies and open-source software.

### Workflow 1—Procopio and Buckley vs S-Trap Protocol

The Millipore ZipTip technology with C18 resin has been routinely used in sample preparation protocols; it allows for desalting, concentration, and purification in a single step, completely removing the lysis solutions used for protein solubilization prior to mass spectrometry analysis.^36^ In fact, the complete removal of salts present in the sample is fundamental in mass spectrometric runs as their presence may create ionic adducts and interfere with the subsequent analysis.^37^ Recently, S-Traps were reported as advantageous over other standard sample preparation methods (FASP and in-solution digestion SP3 beads), out-performing older protocols when comparing the number of unique proteins identified, required protocol times, and reproducibility.^38^

When comparing the relative abundances of shared proteins (*n* = 66) identified using the two protocols in Workflow 1, the Procopio and Buckley group consistently exhibited higher average abundances than the S-Trap group. This disparity may be attributed to protocol differences; notably, the S-Trap protocol necessitates protein quantification during the process, followed by the specific loading of up to 100 μg of proteins into the S-Traps. In contrast, the other workflow does not require protein quantification and does not impose specific limitations on the maximum quantity of proteins to be loaded onto the C18 tip. Ultimately, this results in the normalization of protein concentrations when using the S-Trap protocol, allowing for better intersample and interindividual comparisons and more reliable considerations on the biological variability existing between the samples than the in-StageTip protocol. While other factors could contribute to this result, it is likely that the quantification process plays an important role.

The number of proteins identified (*n* = 109–138 proteins for Procopio and Buckley and for S-Trap GuHCl/Tris protocol, respectively) are consistent and comparable to previous forensic proteomic investigations;^4−6,39^ however, proteins shared between the two protocols were lower in comparison (*n* = 66), due to high missingness values. It also has to be noted that the works previously cited conducted analyses in the DDA mode but using different proteomics software (e.g., Progenesis QI for Proteomics), which allow for the obtainment of less (or no) missing values using their proprietary “unique codetection” approach and overall result in the identification of a higher number of proteins compared with the free software used in this study. In terms of proteome recovery (e.g., the number of different proteins found per protocol), it appears evident that the S-Trap protocol outperforms the in-StageTip one. At the peptide level, multiple peptides that were identified with the S-Trap protocol were not found with the Procopio and Buckley protocol and *vice versa*. Regarding the decreased deamidation observed for the S-Trap group, it is possible that the reduced digestion times required by the S-Trap protocol resulted in less laboratory-induced PTMs, as also suggested in Procopio and Buckley.^14^ However, we cannot make a strong statement regarding the interpretation behind these significant changes in PTM ratios due to the limited amount of peptides shared between the groups.

### Workflow 2—S-Trap Lysis Solutions

Within this part of the study, we compared the S-Trap manufacturer-recommended lysis solution (5% SDS, 8 M urea, 100 mM glycine, pH 7.55) versus Procopio and Buckley buffer (6 M GuHCl, 100 mM Tris, pH 7.40), using S-Trap devices in both cases. Notably, the manufacturer’s buffer has not been specifically optimized for forensic or archeological use on skeletal samples, while the Procopio and Buckley one has specifically been optimized for forensic applications (specifically for chronological estimations).

A similar performance was observed throughout the process when conducting bioinformatics analyses. The similarity of data missingness levels (Figure S3) overall indicates experimental reproducibility. Analyses also showed overlapping modification ratios, as evidenced by PCA and heatmap results, revealing minimal separations between the groups.

The majority of the proteins shared between the two groups (78/112) showed no significant differences in terms of their relative abundances, supporting the lack of striking differences when using these two different solubilization reagents (Table S6.3). However, the PCA conducted at the protein level showed a moderate separation between the two groups, further confirmed by the heatmap where different clusters were noticeable and overall higher relative abundances were achieved for the majority (30 out of 34) of the statistically different proteins when using the SDS buffer. It is important to highlight that analyses were conducted on 30 different individual samples and, therefore, that interindividual biological differences cannot be ignored. As an example, by looking at Figure 5B, it is possible to see a group of samples clustering together despite the lysis buffer used, and represented by lower abundances of any protein, in comparison with the others. This includes samples C19, C23, C22, C27, and C14 (for the SDS treatment) and the respective B19, B23, B22, B27, and B14 (for the GuHCl treatment). For these samples, biological variability may have played a greater role in the lysis solution variation in the overall proteomic abundances. It seems that the SDS buffer may be more effective in solubilizing the proteins still present in the demineralized pellet, despite a slightly (but significant) higher number of (potentially laboratory-induced) deamidations found when using this protocol in comparison with the GuHCl one. Also in this case, the low number of shared peptides identified in this workflow mandates caution when interpreting these statistical evaluations. In principle, both lysis solutions may be appropriate for conducting bone proteomics work with a special emphasis on PMI and age-at-death estimation in forensics. Ultimately, users should carefully consider their research goals to decide which lysis solution may be more appropriate.

### Workflow 3—DDA vs DIA Acquisition Modes

Here, we compared 10 of the 30 samples used in Workflow Two acquired in both DDA and DIA modes. Specific concerns were identified in this experiment; first, there was a notable difference in protein recovery between the two modes (as expected, considering the nature of DIA acquisitions in comparison with DDA ones), and second, the quantification of proteins was less readily comparable due to the fundamentally different data acquisition and different software for sample quantification. Overall, these findings bring into question the reproducibility of the results in different acquisition modes.

There were differences in both the count of unique proteins prior to matching (DIA mode identified 94 more proteins than DDA in terms initially) and missing data after matching; 37 proteins were matched between proteins before being reduced to the final 22 after removal and imputation.

The number of extracted proteins for GuHCl/Tris in the DIA mode in Workflow Three (*n* = 143) was higher than the number obtained in Workflow One and Workflow Two (*n* = 138) due to the reduced amount of samples being processed in the preprocessing stage specifically (*n* = 10 versus *n* = 30). It is noteworthy that the observed differences between the DDA and DIA modes in terms of protein relative abundances are based on a relatively small number of proteins (*n* = 22); therefore, trends identified here should be considered with caution.

Multivariate analysis revealed distinct clustering between the groups at the protein level. Notably, collagenous and collagen-binding proteins observed (COL1A2, COL1A1, COL6A1, and COL6A3) exhibit the lowest variance across the modes. Collagenous proteins typically dominate the analytical space in the bone proteome among different species, with noncollagenous proteins having a lower-abundance and greater variability;^40,41^ however, for previous forensic proteomic investigations, there has been a greater focus on the noncollagenous proteins due to their longevity and survivability in decayed remains being present in the inorganic hydroxyapatite of the bone.^5,6,19^

In comparison, some of the noncollagenous proteins showed a greater variance between the two acquisition modes. The DDA mode is generally more suited to detecting higher abundance proteins because it focuses on selecting and fragmenting the most abundant ions from each survey scan. This means it is well suited for protein identification, but there are challenges in accurate quantification, especially for low-abundance proteins due to the stochastic nature of precursor ion selection. Conversely, DIA is less biased toward highly abundant peptides, allowing for a more comprehensive sampling of the peptide population. This can be highly advantageous for quantitative proteomics as it systematically fragments all peptides, providing a more consistent and reproducible measurement of peptide abundance. Interestingly, proteins such as tropomyosin, hemoglobin, desmin, heat shock proteins, filamin, and prolargin were found to be more abundant in DDA acquisition modes, whereas others such as vitronectin, decorin, and proteolipid protein 1 were found to be more abundant in the DIA mode. As observed for the lysis solution comparison, also in this case, it was possible to identify a sample, the number “1” located at the left-hand side of the heatmap (Figure 7B), characterized by relative abundance levels different from all of the other samples and not necessarily related with the acquisition mode used.

Given the higher abundance and narrow variability of collagenous proteins in human bone, both the DDA and DIA modes offer a more effective assessment of their relative abundance compared to the less abundant noncollagenous proteins (despite COL1A2 and COL6A1 exhibiting significant differences in their abundances between the two modes, with DDA generating less abundant values than DIA). However, limitations of DDA workflows arise from its dynamic range, saturation with high-abundance peptides, and potentially under-representation of low-abundance peptides, as observed in our data (Figure 7B). In contrast, DIA has a greater dynamic range, making it more suitable for capturing information from both high- and low-abundance peptides from a single experiment, leading to greater reproducibility compared to DDA. Therefore, there are several factors to consider in what may drive the overall difference between the modes of interest (DDA and DIA) ranging from these highlighted dynamic range, peptide selection, and sample complexity factors.^42−44^ Additionally, this variability between runs and differences in peptide identification and quantification align with the known issues of reproducibility in DDA experiments, attributed to stochastic precursor ion selection. However, the DIA mode can often produce more reproducible results as it consistently targets all precursor ions within a specified mass range, offering better consistency and reproducibility of protein identification compared to the DDA mode.^45,46^ Moreover, the ability of the DIA mode to offer deep coverage and quantification of the bone proteome provides enhanced opportunities for new discoveries in skeletal biology and disease, which is pertinent in forensic biomarker applications.^47^

In terms of disadvantages of DIA, it is essential to recognize that in complex samples, the application of DIA may introduce uncertainty and, therefore, not perform with the equivalent level of confidence as other techniques such as DDA.^48^ For example, within a complex sample, it is possible that other interfering compounds may share the same mass as other ions, potentially leading to greater false positive rates.^49^ It is also pertinent to acknowledge that while DDA enables a more targeted and confident identification of peptides and proteins, the broader and less selective nature of DIA may result in identifications that do not hold the same level of certainty, which is particularly critical in forensic investigations where accuracy and reliability are paramount. Furthermore, the indiscriminate nature of DIA can lead to increasing complexity in data interpretation, potentially complicating forensic analysis. Such challenges can be largely attributed to differences between MS and MS/MS spectra, i.e., tracing shared fragments derived from coisolated precursor ions.^50,51^ Since DIA uses a wider precursor isolation window compared to DDA, contaminant peptides in DIA are more likely to be coeluted and cofragmented with other peptides, potentially resulting in false identification of peptides/proteins.^52^ When comparing MS2 spectra between both the DIA and DDA modes, it has been demonstrated that spectra quality is generally higher in the DDA mode, but the number of spectra is generally greater in the DIA mode.^52^

### User Experience and Workflow Decision

Selecting an optimal workflow depends on the user’s specific requirements. For achieving a comprehensive identification of proteins and peptides in an untargeted high-throughput approach, the integration of S-Traps with the DIA mode emerges as a favored choice. However, this does not negate the use of alternative workflows as their selection depends on many factors including sample size, ultimate aims of the study, instrument availability, and type of samples.

For instance, when the sample count is large (e.g., *n* > 100), ZipTips might be preferred since the assessment and selection of protein quantity before S-Trap loading can be labor-intensive if dealing with a diverse range of sample quantities. However, these steps ensure normalization of the results, ultimately making the S-Trap protocol recommended for increased reproducibility and reduced user errors. It is worth noting the issue of samples drying when full plates are loaded, which can be mitigated by covering the loaded wells with strip caps. Moreover, the use of a large quantity centrifuge is pivotal in the S-Trap micro protocol, which employs tubes instead of 96-well plates due to limitations related to the minimum desired protein quantity. While S-Trap plates exist, they have specific protein quantity requirements (100–300 μg), which are hindered by the variability in quantity from archeological and forensic samples due to taphonomic alteration over time. When the tubes are used, the procedure can be expedited by piercing the tubes and placing the filters on top of the centrifuge. Following this, loading the desired washing buffers directly into the filter in the centrifuge without opening or closing the lids every time can enhance efficiency. On the other hand, ZipTips require carefully trained pipetting to avoid batch effects. The Procopio and Buckley protocol highlights additional challenges when using molecular weight-cutoff (MWCO) tubes with specific lysis solutions (e.g., EDTA), causing delays in subsequent steps, as frequently samples do not pass through the filter at the same speed.

The comparison of DDA and DIA highlights specific functional differences. Rather than establishing superiority, the optimal choice hinges on accessibility and experimental objectives as well as instrument and software availability. It is uncommon to find high-resolution instruments required to conduct DIA analyses in crime laboratories, although they may be more readily available in forensic toxicology settings. This may result in the limited immediate applicability of such protocols in forensic settings. However, the DIA acquisition mode is becoming more explored in forensic and archeological scenarios, due to its ability to provide robust identifications for the analytes of interest, including low-abundant proteins and providing enhanced reproducibility; examples include the use of DIA to analyze genetically variant peptides (GVPs), to conduct species identification of archeological bones, and to perform species authentication for food fraud.^27,53,54^

Researchers may select a specific method (DDA or DIA) depending on the software options available for data analysis and the ease of use offered by the mass spectrometry facility. In this study, we compared DIA-NN and MaxQuant; software choices may affect the proteins/peptides identified; however, the software-dependent differences between the DIA and DDA results would most likely be minor. For identifying post-translational modifications (PTMs) and greater proteome coverage through untargeted approaches, DIA analysis is the recommended workflow. However, if the focus is on comparing relative abundances at the protein level based on previous investigations, the DDA protocol used by Procopio and Buckley may be more suitable.

## Conclusions

Overall, it is vital to define optimal processing in the growing field of forens-omics, focusing on methods that are minimally destructive, easy to perform, and with robust extraction and reproducible measurement. Our data suggest that an integrated approach between the S-Traps and DIA modes will undoubtedly improve future biomolecular investigations in bone tissue and may be used in forensic research and caseworks.

## Data Availability

The mass spectrometry proteomics data have been deposited to the ProteomeXchange Consortium via the MASSIVE^55^ partner repository with the data set identifier MSV000093803.

## Supporting Information Available

Protein extraction protocols tested in the workflows (Table S1); material sources choosing an imputation algorithm; flowchart for imputation algorithm decision (Figure S1); missingness reported for Workflow One (Figure S2); missingness reported for Workflow Two (Figure S3); missingness reported for Workflow Three (Figure S4); performance metrics for each tested algorithm in Workflow One (Table S2); performance metrics for each tested algorithm in Workflow Two (Table S3); performance metrics for each tested algorithm in Workflow Three (Table S4); choosing a missingness (%) cutoff threshold proteome coverage; Workflow One PTMs heatmap (Figure S5); Workflow Two PTMs heatmap (Figure S6) (PDF)
Coverage data and statistical tests (Table S5); average coverage results for Workflow One proteins (Table S5.1); Welch T-Test for Workflow One coverage results (Table S5.2); average coverage results for Workflow Two proteins (Table S5.3); Welch T-Test for Workflow Two coverage results (Table S5.4); average coverage results for Workflow Three proteins (Table S5.5); Welch T-Test for Workflow Three coverage results (Table S5.6) (XLSX)
Wilcoxon-signed rank results for Workflow One proteins (Table S6.1); Wilcoxon-signed rank results for Workflow One peptides; MOD1 refers to the deamidation specific PTM, MOD2 refers to the oxidation specific PTM (Table S6.2); Wilcoxon-signed rank results for Workflow Two proteins (Table S6.3); Wilcoxon-signed rank results for Workflow Two peptides; MOD1 refers to the deamidation specific PTM, MOD2 refers to the oxidation specific PTM (Table S6.4); Wilcoxon-signed rank results for Workflow Three proteins (Table S6.5) (XLSX)
