# Supplementary material for: Bone Proteomics Method Optimization for Forensic Investigations
Source: J Proteome Res. 2024 Apr 15;23(5):1844–58. doi: 10.1021/acs.jproteome.4c00151 (PMC11077585; doi:10.1021/acs.jproteome.4c00151)
Supplement: Supplementary file 1 — pr4c00151_si_002.pdf [file pr4c00151_si_002.pdf]

Supplementary Materials:

Table S1 – Protein extraction protocols tested in the workflows.

Procopio and Buckley protocol:	Adapted 'S-Trap' protocols:
1. Decalcification:<br>• 25mg bone powder, 1mL 10% FA for 6h at +4°C. Acid-soluble fraction separated and frozen.<br>2. Lysis:<br>• Acid-insoluble pellet, 500µL 6M GuHCl/100mM Tris pH 7.4 for 18h at +4°C.<br>3. Buffer exchange:<br>• Acid-insoluble fraction exchanged with 50mM AMAC.<br>4. Reduction:<br>• 5mM DTT for 45 min at R/T.<br>5. Alkylation:<br>• 15mM IAM for 40 min at R/T in the dark.<br>6. Quenching:<br>• 5mM DTT.<br>7. Digestion:<br>• 1µg trypsin for 5h at +37°C.<br>8. Acidification:<br>• 1 v/v % TFA<br>9. Purification, desalting and concentration:<br>• ZipTip® following manufacturer's instructions. Wash in 0.1 v/v % TFA, elution in 50 v/v % ACN/0.1 v.v% TFA.	1. Decalcification:<br>• 25mg bone powder, 1mL 10% FA for 6h at +4°C. Acid-soluble fraction separated and frozen.<br>2. Lysis:<br>• Acid-insoluble pellet, 500µL 6M GuHCl/100mM Tris pH 7.4 for 18h at +4°C **or** 500uL 5% SDS, 8M urea 100mM glycine pH 7.5 (depending on which workflow) for 18h at +4°C.<br>3. Quantification:<br>• Pierce™ BCA Protein Assay Kit.<br>• Aim for 50µg of protein in final volume of 25µL.<br>4. Reduction:<br>• 5mM DTT for 45 min at R/T.<br>5. Alkylation:<br>• 15mM IAM for 40 min at R/T in the dark.<br>6. Quenching:<br>• 5mM DTT.<br>7. Acidification:<br>• 2.5µL 55% phosphoric acid (Sigma-Aldrich, U.K.)<br>8. Trap and wash:<br>• 165µL binding/wash buffer (90 v/v % MeOH and 10 v/v % TEAB). Loading of samples on S-Trap™ and centrifugation. 150µL of binding/wash buffer added three other times.<br>9. Digestion:<br>• 10µg trypsin for 2h at +47°C.<br>10. Elution:<br>• 40µL TEAB 50mM<br>• 40µL 0.1 v/v % FA<br>• 40µL 50% ACN/0.1 v/v % FA

Material Sources:

Formic Acid - Sigma Aldrich, U.K.
Guanidine hydrochloride 6M/ Tris buffer 100 mM (pH 7.4) - Sigma-Aldrich, U.K.
SDS (5%), 8M urea 100mM glycine at (pH 7.5) -(Sigma-Aldrich, U.K.
Ammonium acetate 50 mM - Scientific Laboratory Supplies, U.K.

Dithiothreitol (DTT) - Fluorochem, U.K.
Samples quantified through the 2D Quant Kit - Sigma Aldrich U.K.
Iodoacetamide (IAM) - Sigma-Aldrich, U.K.
Phosphoric acid - Sigma-Aldrich, U.K.
Methanol Absolute - Sigma-Aldrich, U.K.
Tetraethylammonium bromide (TEAB) (50mM) - Sigma-Aldrich, U.K.
Acetonitrile (ACN) in 0.1% Formic acid Sigma Aldrich, U.K.
Trypsin - Promega, U.K.
Trifluoroacetic acid (TFA) - Fluorochem, U.K.
OMIX C18 Zip-tip - Agilent Technologies, U.S.A.

Choosing an imputation algorithm:
An optimal imputation algorithm was identified by testing four imputation methods commonly applied to proteomic data: Random Forest (MissForest Package v.1.5(Stekhoven & Bühlmann, 2012)), Interpolation (ImputeTS Package v.3.3) (Moritz & Bartz-Beielstein, 2017), Lasso linear regression (MICE Package v.3.16) and arithmetic mean (MICE Package v.3.16) (van Buuren & Groothuis-Oudshoorn, 2011).

Random-forest imputation is commonly used in proteomic workflows since it gives consistent and reliable performance with a variety of datasets (Egert et al., 2021; Jin et al., 2021; Stekhoven & Bühlmann, 2012). However, it was compared to the above methods to validate its performance with the different workflow datasets in this investigation.

For each workflow, proteins were matched between comparator datasets. Missingness is summarised in Figures S2, S3 and S4 for workflows one, two and three, respectively. Artificially missing data was introduced for each workflow to compare imputation methods (Figure S1). Random forest imputation outperformed the other techniques for each workflow (Tables S2, S3 and S4) and was used thereafter to impute missing data. For a given protein or peptide, imputation was performed if <50% (protein) or <35% (peptide) data was missing across the cohort. These cut-offs were empirically determined by artificially removing data (from 30%-50% at 5% intervals) and assessing concordance between true and imputed data values.

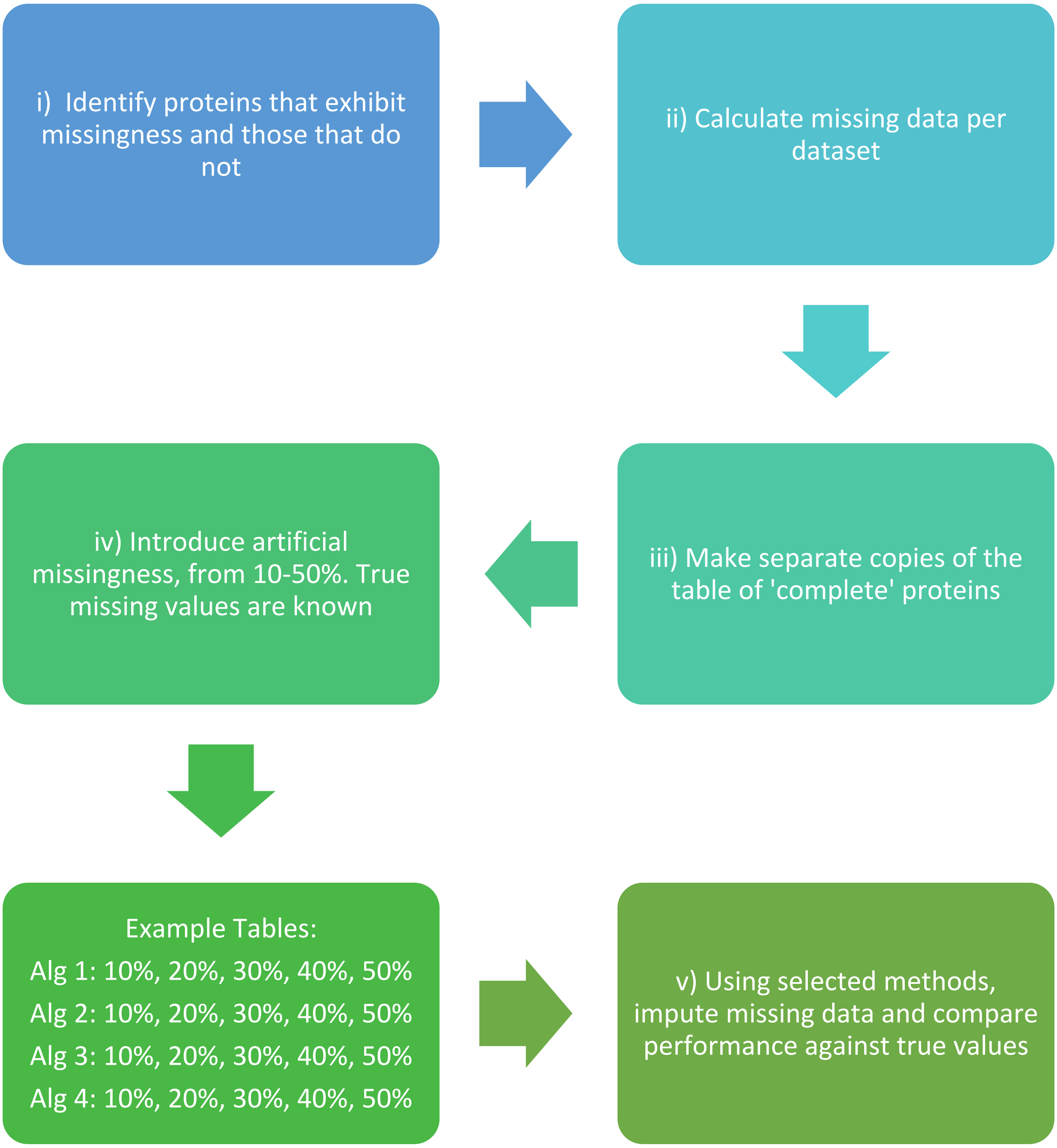

Fig. S1 – *This flowchart establishes the roadmap for investigating each workflow dataset and identifying which imputation algorithm could handle increasing levels of missingness similar to that of the original datasets. The limit of 50% was chosen as the maximum missingness to be tested, as the authors agree anything beyond 50% missing should be removed and should not be attempted to undergo imputation.*

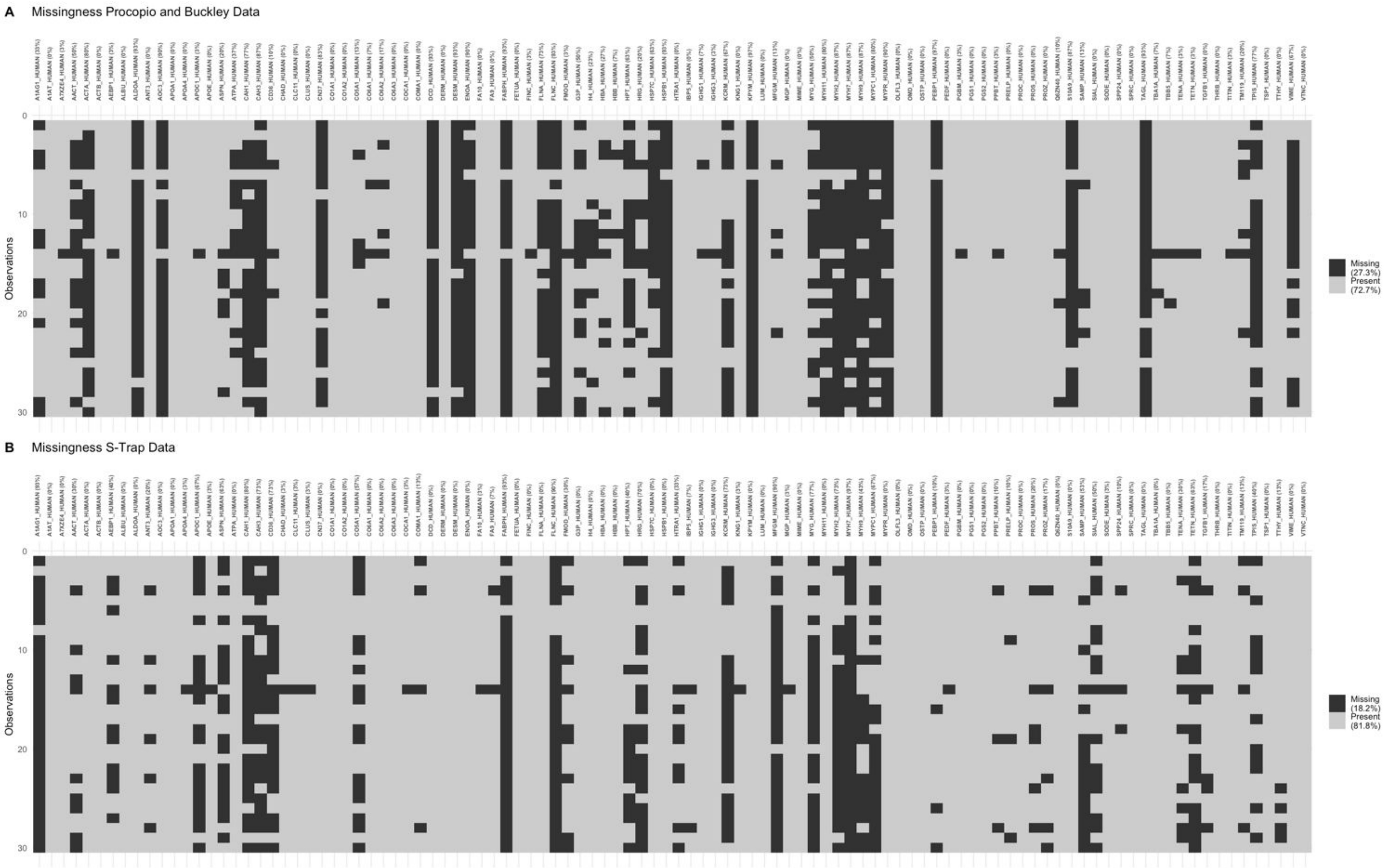

Fig. S2 (A) – Missingness reported for Procopio and Buckley dataset of Workflow One (B) – Missingness reported for S-Trap dataset of Workflow One.

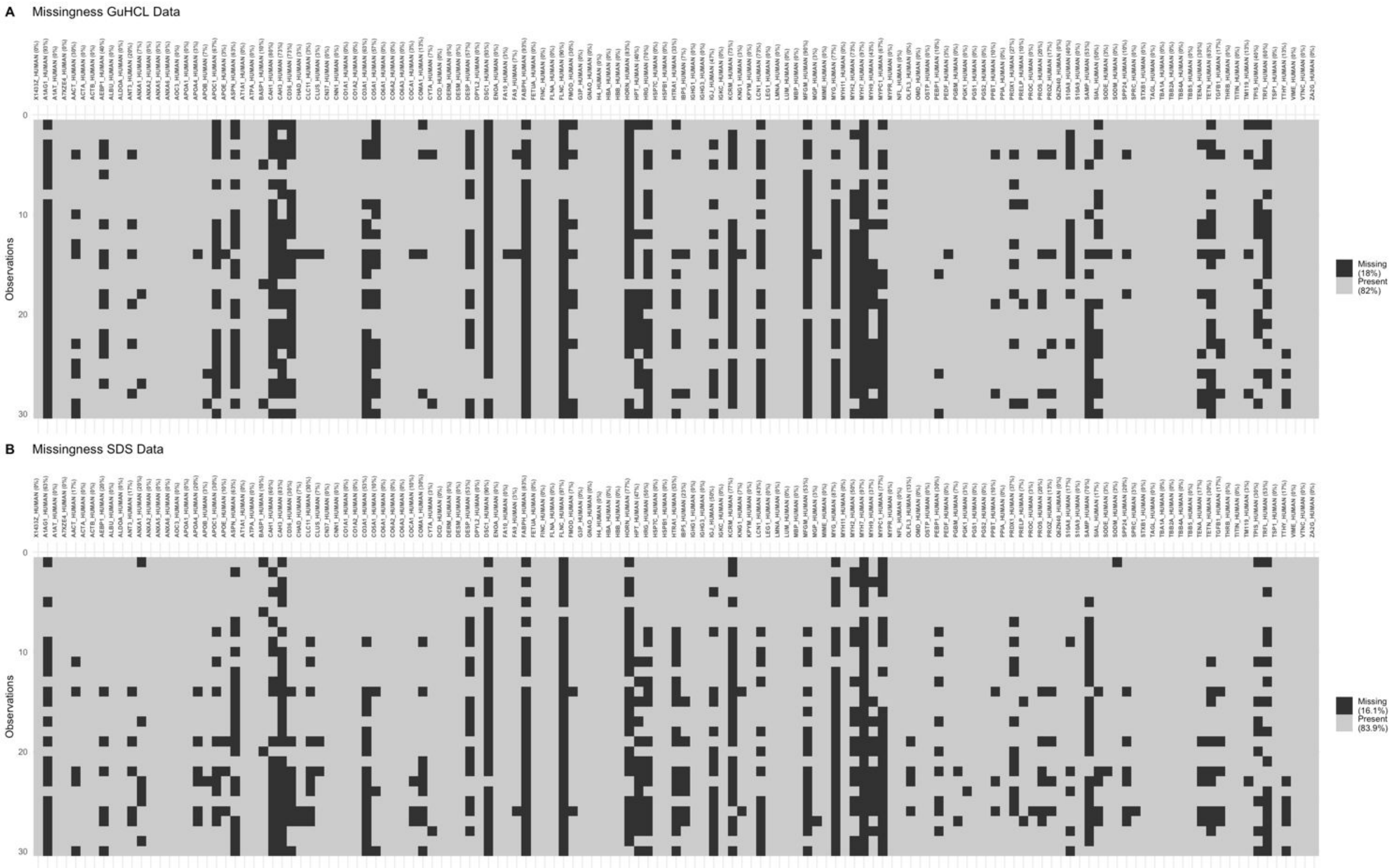

Fig. S3 (A) – Missingness is reported for GuHCl dataset of Workflow Two. (B) – Missingness is reported for SDS dataset of Workflow Two

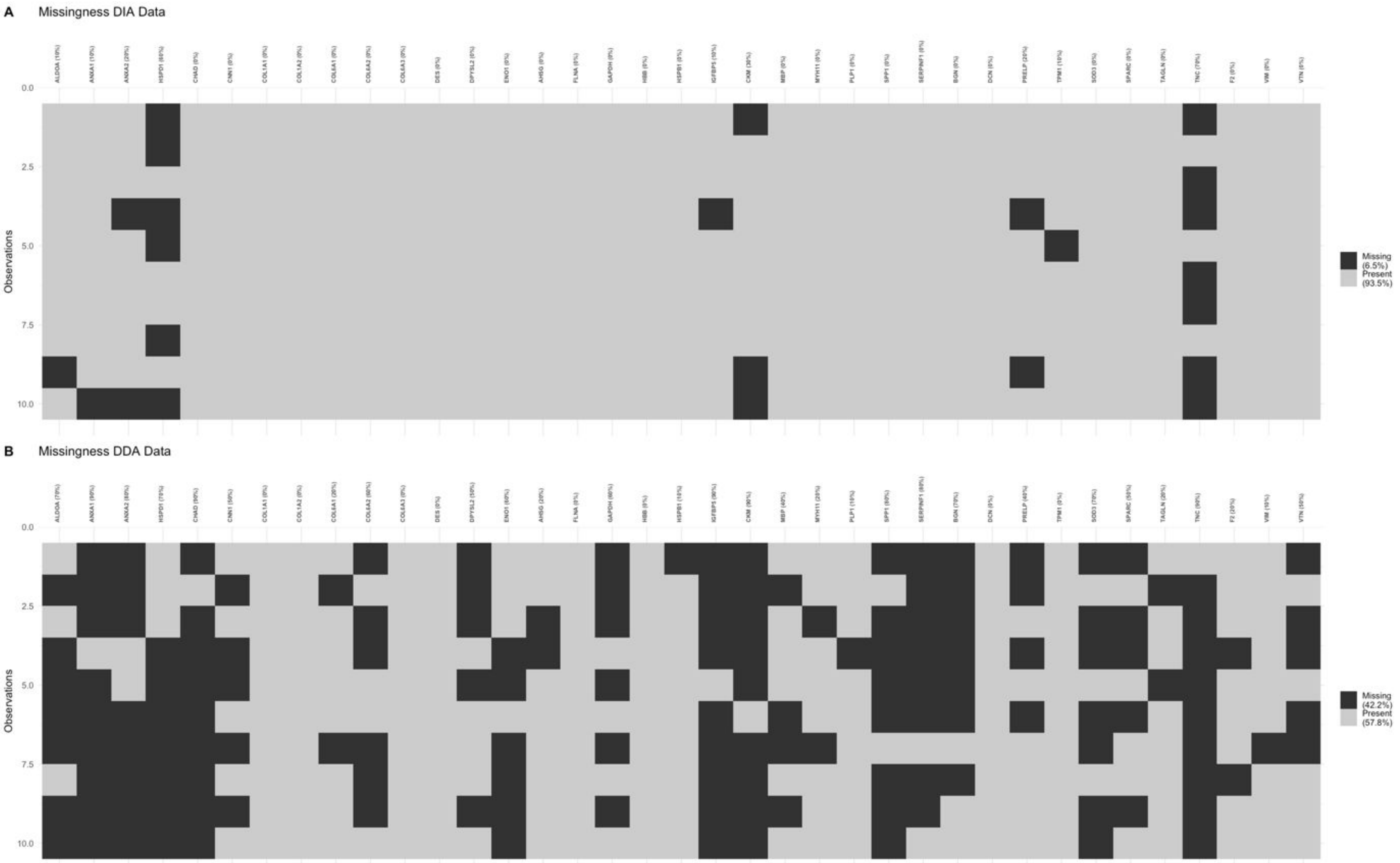

Fig. S4 (A) – Missingness is reported for DIA dataset of Workflow Three. (B) – Missingness is reported for DDA dataset of Workflow Three.

Table S2 – Table shows the performance metrics (RMSE and Cor scores) for each tested algorithm in Workflow One. PB is Procopio and Buckley. RMSE is Root Mean Square Error. "Cor" is the Pearson Correlation coefficient.

Workflow/Group	Algorithm	RMSE (Normalised Abundance)	Cor
One/PB	Lasso	1.08	0.50
One/PB	Mean	1.29	- 0.15
One/PB	MissForest	1.04	0.56
One/PB	TimeSeries	1.30	0.23
One/S-Trap	Lasso	0.78	0.46
One/S-Trap	Mean	0.82	- 0.20
One/S-Trap	MissForest	0.64	0.59
One/S-Trap	TimeSeries	0.89	0.20

Table S3 - Table shows the performance metrics (RMSE and Cor scores) for each tested algorithm in Workflow Two. PB is Procopio and Buckley. RMSE is Root Mean Square Error. "Cor" is the Pearson Correlation coefficient.

Workflow/Group	Algorithm	RMSE (Normalised Abundance)	Cor
Two/GuHCl	Lasso	0.83	0.39
Two/GuHCl	Mean	0.81	-0.21
Two/GuHCl	MissForest	0.66	0.53
Two/GuHCl	TimeSeries	0.82	0.23
Two/SDS	Lasso	0.90	0.50
Two/SDS	Mean	1.06	-0.18
Two/SDS	MissForest	0.75	0.64
Two/SDS	TimeSeries	0.96	0.43

Table S4 - Table shows the performance metrics (RMSE and Cor scores) for each tested algorithm in Workflow Three. PB is Procopio and Buckley. RMSE is Root Mean Square Error. "Cor" is the Pearson Correlation coefficient.

Workflow/Group	Algorithm	RMSE (Normalised Abundance)	Cor
Three/DIA	Mean	1.04	- 0.41
Three/DIA	MissForest	0.97	- 0.15
Three/DIA	TimeSeries	1.06	- 0.05
Three/DDA	Mean	1.43	- 0.38
Three/DDA	MissForest	1.39	- 0.13
Three/DDA	TimeSeries	1.67	0.11

Choosing a Missingness (%) cut-off threshold:

The missingness (%) cut-off threshold was decided based on comparing the distributions for protein normalised abundances and peptide modification ratios per workflow. They were compared within increasing imputation thresholds from 30%-50% in 5% intervals. There were no major changes to the distribution of proteins after imputation up to 50% missingness; for peptides, changes in the distribution of peptides were evident above 35%. Therefore the missingness (%) cut-off threshold for proteins was set at 50% and for peptides at 35%;

proteins/peptides above their respective missingness cutoff were removed from further consideration.

Proteome Coverage

Coverage in these studies was defined as the proportion of unique identifiable peptide sequences among the identified proteins, compared to the entire database reference sequences.

All proteins prior to missingness cleaning, as well as non-proteotypic proteins and those with <2 unique peptides were included. Coverage was calculated at the individual sample level and per group. Proteins between each workflow were matched to compare coverage per protein of interest.

Statistical analysis was conducted on the normalised coverage values using a Welch T-Test for both a group comparison and per protein comparison (Table S5).

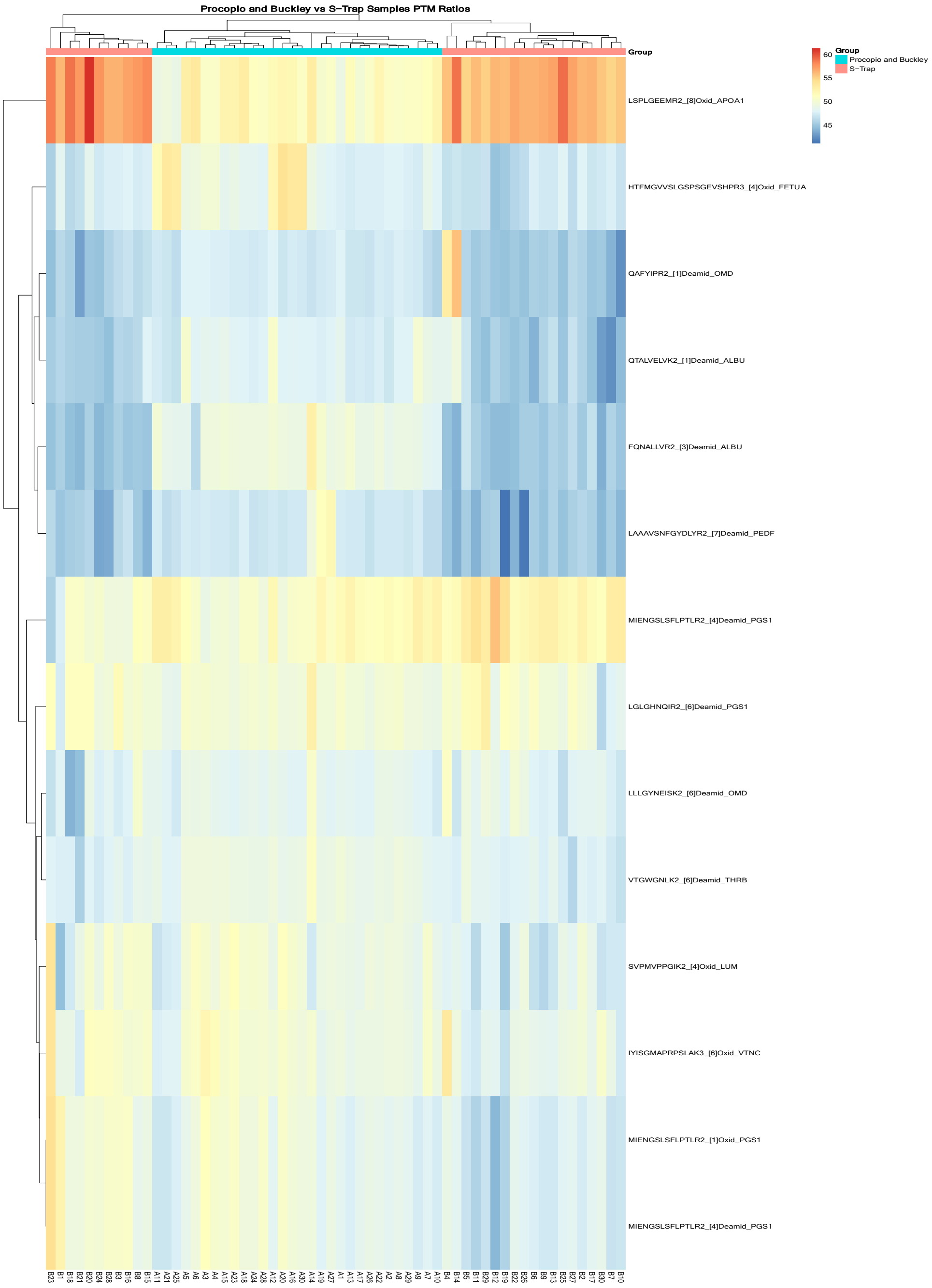

Fig. S5 – Heatmap with Euclidean hierarchical clustering between workflow one subgroups ‘Procopio and Buckley’ and ‘S-Trap’ for modified peptides. Scale is in modification ratio percentage (%).

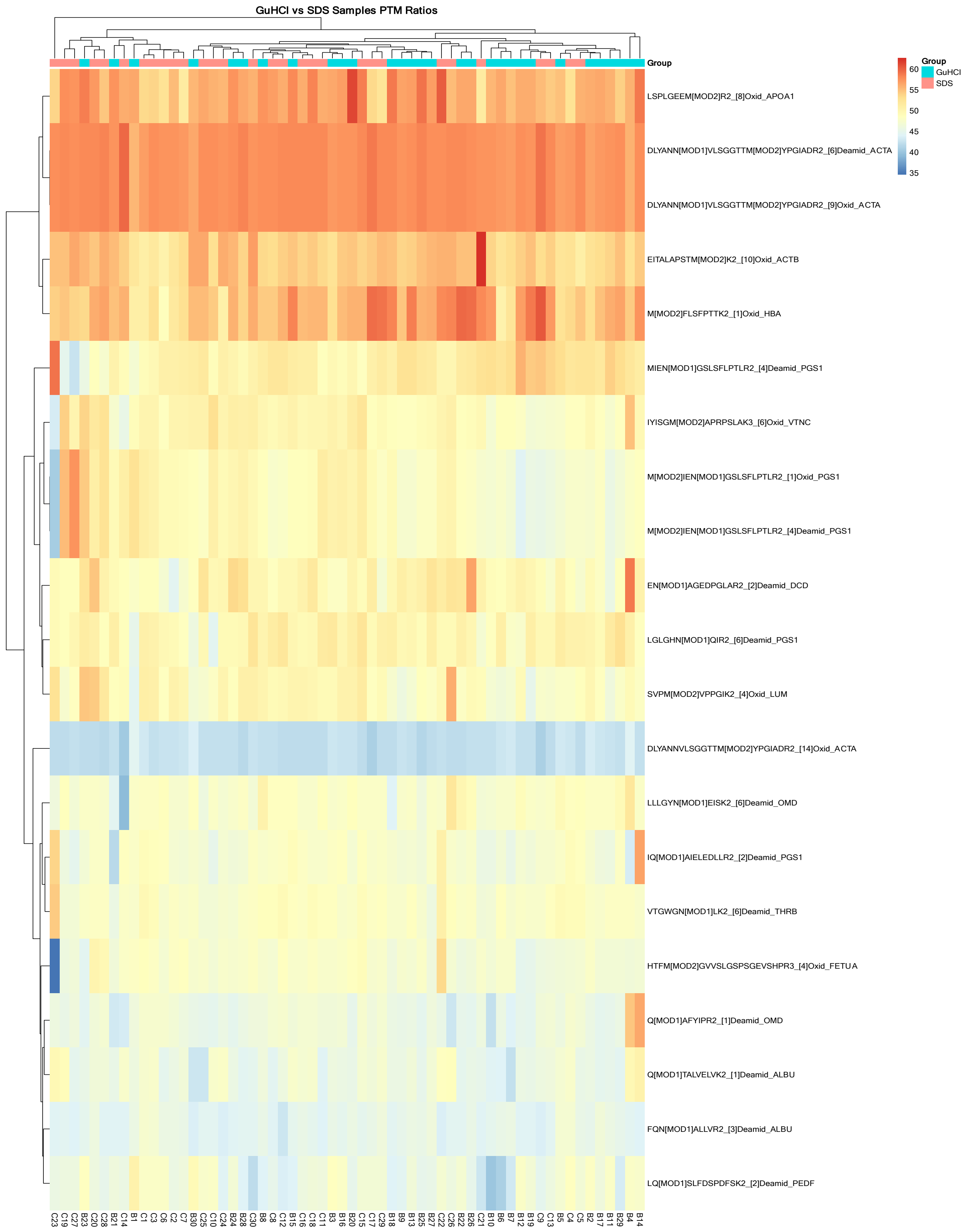

Fig. S6 - Heatmap between workflow one subgroups 'GuHCl' and 'SDS' for modified peptides. Following the sequence is the position of the modification, the modification itself and the protein. Scale is in modification ratio percentage (%).

References:

Egert, J., Brombacher, E., Warscheid, B., & Kreutz, C. (2021). DIMA: Data-Driven Selection of an Imputation Algorithm. *Journal of Proteome Research*, *20*(7), 3489–3496. https://doi.org/10.1021/acs.jproteome.1c00119

Jin, L., Bi, Y., Hu, C., Qu, J., Shen, S., Wang, X., & Tian, Y. (2021). A comparative study of evaluating missing value imputation methods in label-free proteomics. *Scientific Reports*, *11*(1). https://doi.org/10.1038/s41598-021-81279-4

Moritz, S., & Bartz-Beielstein, T. (2017). imputeTS: Time series missing value imputation in R. *R Journal*, *9*(1), 207–218. https://doi.org/10.32614/rj-2017-009

Stekhoven, D. J., & Bühlmann, P. (2012). Missforest-Non-parametric missing value imputation for mixed-type data. *Bioinformatics*, *28*(1), 112–118. https://doi.org/10.1093/bioinformatics/btr597

van Buuren, S., & Groothuis-Oudshoorn, K. (2011). mice: Multivariate imputation by chained equations in R. *Journal of Statistical Software*, *45*(3), 1–67. https://doi.org/10.18637/jss.v045.i03
